# Inequalities in local government spending on cultural, environmental and planning services: a time-trend analysis in England, Scotland, and Wales

**DOI:** 10.1186/s12889-023-15179-9

**Published:** 2023-02-28

**Authors:** Katie Fahy, Alexandros Alexiou, Kate Mason, Davara Bennett, Matt Egan, David Taylor-Robinson, Ben Barr

**Affiliations:** 1 Department of Public Health, Policy and Systems, University of Liverpool, Liverpool, L69 3GB UK; 2 Centre for Health Policy, Melbourne School of Population and Global Health, The University of Melbourne, Melbourne, Australia; 3 Department of Public Health, Environments and Society, London School of Hygiene and Tropical Medicine, London, UK

**Keywords:** Health inequality, Local government, Austerity, Culture, Environmental health, Planning

## Abstract

**Background:**

Local government provides Cultural, Environmental, and Planning (CEP) services, such as parks, libraries, and waste collection, that are vital for promoting health and wellbeing. There have been significant changes to the funding of these services over the past decade, most notably due to the UK government’s austerity programme. These changes have not affected all places equally. To understand potential impacts on health inequalities, we investigated geographical patterning of recent CEP spending trends.

**Methods:**

We conducted a time trend analysis using routinely available data on local government expenditure. We used generalised estimating equations to determine how expenditure trends varied across 378 local authorities (LAs) in Great Britain between 2009/10 and 2018/19 on the basis of country, deprivation, rurality, and local government structure. We investigated the gross expenditure per capita on CEP services, and the CEP expenditure as a proportion of total local authority budgets. We present the estimated annual percentage change in these spend measures.

**Results:**

Expenditure per capita for CEP services reduced by 36% between 2009/10 and 2018/19. In England, the reduction in per capita spending was steepest in the most deprived quintile of areas, falling by 7.5% [95% CI: 6.0, 8.9] per year, compared to 4.5% [95% CI: 3.3, 5.6] per year in the least deprived quintile. Budget cuts in Scotland and Wales have been more equitable, with similar trends in the most and least deprived areas. Welsh LAs have reduced the proportion of total LA budget spent on CEP services the most (-4.0% per year, 95% CI: -5.0 to -2.9), followed by Scotland (-3.0% per year, 95% CI: -4.2 to -1.7) then England (-1.4% per year, 95% CI: -2.2 to -0.6). In England, rural and unitary LAs reduced their share of spending allocated to CEP more than urban and two-tier structured LAs, respectively.

**Conclusion:**

Funding for cultural, environmental and planning services provided by local government in the UK has been cut dramatically over the last decade, with clear geographical inequalities. Local areas worst affected have been those with a higher baseline level of deprivation, those with a single-tier local government structure, and English rural local authorities. The inequalities in cuts to these services risk widening geographical inequalities in health and wellbeing.

**Supplementary Information:**

The online version contains supplementary material available at 10.1186/s12889-023-15179-9.

## Introduction

In the last decade, there have been significant changes to public spending in the UK, especially for local government. Since 2010, most departments of government have experienced cuts in funding as a result of the government’s austerity programme that sought to reduce the public funding deficit that arose following the financial crisis of 2008 [1]. However, of all departments, the local government department has been one of the most significantly affected. Funding cuts have required local government to dramatically restructure budgets to continue to meet population needs. Local government, termed ‘local authorities’ in the UK, provide vital services for their resident populations including social care, housing, transport, Cultural, Environmental and Planning (CEP) services. CEP services have been subject to the greatest spending cuts of all areas of local government provision, with spending on planning and development services reducing in real terms by 42% between 2009 and 2018, compared to a 5% reduction in spending on social care services over the same period.

The specific budget lines of CEP expenditure include: cultural and related services, such as parks, leisure centres, libraries, museums and art galleries; environmental and regulatory services, such as waste collection, food and water safety, community safety; and planning and development services, such as monitoring and enforcing building regulations, planning policy, economic and community development [2–4]. These services provide an avenue for public investment in place-based social determinants of health. Particularly when implemented as part of greater efforts to reduce economic inequalities [5], place-based approaches are widely recognised as effective for improving population health and health equity [6, 7]. There are many potential pathways whereby CEP services enhance the health and well-being of the communities that they provide for by influencing determinants of health such as the built environment, communities and activities [8]. Many services are free or subsidised at the point of use: an important mechanism for benefiting residents on lower incomes.

Cultural and related services made up approximately 28% of the CEP budget in financial year 2018/19. These services are likely to impact population health through a number of pathways. Multiple systematic reviews have evidenced the importance of greenspace for the mental health of children, through pathways such as moderating stress and improving issues with hyperactivity and inattention [9, 10]. Mental health benefits have also been found in adults [11], as well as other benefits important for health and well-being, such as increased physical activity [12] and social cohesion [13]. Physical activity may also be facilitated by leisure centres provided by local authorities [14]. Public libraries, museums and art galleries have also been shown to improve social cohesion [15, 16]. Services such as libraries provide additional benefits to more deprived communities, such as educational opportunities, reduced digital exclusion, a warm safe place to spend time, and assistance with job applications. These additional benefits can be viewed from a wider social determinants of health perspective (education, digital access, warmth, safety, employment) [17].

Environmental and regulatory services accounted for 52% of the CEP budget in 2018/19. These services were founded by the sanitation policies of the Public Health Act 1848, which transformed the public’s health and established local government’s involvement in public health issues. Since then, the services have continued to play a vital role in managing environmental health hazards, such as air and noise pollution [18, 19], infectious diseases, such as food poisoning [20], and regulating industries, such as alcohol licensing policies [21, 22]. Local authorities are required to provide a number of environmental services, for example, services such as street cleansing are necessitated by the Environmental Protection Act of 1990 which states that neighbourhoods must meet a certain level of cleanliness [23]. Environmental services are especially important in this context as there was already evidence prior to the budget cuts that poorer neighbourhoods do not receive sufficient levels of these services to address their needs [24], a problem that may have been exaggerated by reduced resources.

Planning and developmental services cover a range of community and economic development plans, as well as town planning, business support and enforcing building regulations. In 2018/19, they accounted for 20% of the CEP budget. These planning services have a great influence over residents’ environments, impacting levels of active travel, outdoor recreational activity, and accessibility of local social networks [25]. Planning services have important implications for the health, well-being, and social cohesion of neighbourhoods [26]. Therefore, changes in the level and geographical pattern of funding for these services will potentially have an impact on health and health inequalities.

Funding for local government services comes from four main sources: central government grants; fees and service charges; business rates, a local property tax on businesses; and council tax, a local property tax on households. While the mechanisms for allocating central government grants vary between England, Scotland, Wales (see appendix 1), across all countries, central government grants account for the largest proportion of local authority funding. The amount each council receives through grants is determined by their country’s central government, though all countries employ a similar method of assessing relative needs and resources of councils. This assessment is made using formulae that account for indicators of need, such as local demographics, and indicators of resource, such as the council tax base of the local authority, in comparison with others in the country [27, 28]. There are separate formulae for different services (e.g. CEP, social care, etc.). These formulae are currently being reviewed by the government to simplify local government resource allocation in England [29]. One of the proposed changes is that funding for CEP services and similar ‘foundation’ services should be funded on a per capita basis, as opposed to accounting for population characteristics such as level of deprivation. This change would adversely affect more deprived areas, which are given greater weight in the existing formula [30].

Councils then decide on how they will allocate these resources across different service lines. Some services are mandatory, meaning councils have a statutory duty to provide them. For example, many social care services that provide for vulnerable populations. The provision of CEP services is largely discretionary, with the exception of environmental services. This means that when the overall budget for a council is reduced, CEP services may be cut to a greater extent than, for example, social care, so those resources can be used to maintain the provision of these priority statutory services.

Furthermore, latitude in how resources can be redirected between service lines varies between types of local government that have responsibilities for different services. In Scotland and Wales, all municipal authorities are single-tier, or unitary, meaning that they are responsible for all local government functions within their boundaries. In England, some authorities are unitary, while others have a two-tier system in which responsibilities are split between an upper tier (e.g. county) and lower tiers (e.g. districts within a county). Unitary authorities are typically urban areas, such as cities, which are large enough to function independently, while two-tier local authorities are generally more rural. A critical difference in administration between the two systems is that lower-tier local authorities have responsibility for CEP services and upper-tier local authorities have responsibility for other services, such as social care. Therefore, in unitary areas of England it is possible for councils to cut CEP services more to support e.g., social care, whilst in two-tier areas this is much more difficult to do. As of 2018, there are 326, 32, and 22 local authorities in England, Scotland and Wales, respectively. In England, 124 are unitary authorities and 202 are lower-tier local authorities, and in recent years there has been a trend of local authorities moving to a single-tier structure [31].

Understanding how reductions in CEP spending differ across local areas on the basis of their characteristics is an important first step in order to assess potential impacts on health inequalities. Although the decline in spending is almost ubiquitous, there is significant variation in the level of cuts between local authorities. During the study period, in addition to the massive funding cuts, there have been major changes in how funding is allocated to local government; how local government can raise revenue through local taxation, fees, and capital; and individual local authority prioritisation decisions given the uncertainty about future funding [32].

This study sets out to identify whether there are systematic patterns of local government spending trends on CEP services in relation to deprivation, rurality, or local government structure; and to what extent the devolution of Scotland and Wales’ local government affects spending patterns. We will assess these spending patterns using two analyses. The first will assess trends in CEP spending per person, while the second will assess trends in the proportion of total services expenditure allocated to CEP services. The aim of these analyses is to determine the extent to which variation in spending trends is due to differences in overall funding available or due to differences in local resource allocation decisions.

## Methods

### Setting

We conducted a time-trend analysis at local government area level in the UK. We analysed annual expenditure data from 378 lower- and single-tier local authorities in Great Britain (England, Scotland, and Wales), between 2009/10 and 2018/19, based on 2018 geographical boundaries [appendix 2 for full details]. We excluded the City of London (a London area dominated by business premises) and the Isles of Scilly because of their special nature and small population sizes. Finally, we excluded Northern Ireland due to limited data availability.

### Data and measures

The variables of interest in our study are the annual gross expenditure per capita on CEP services and the annual gross expenditure on CEP services as a share of total gross service expenditure on all local authority services, for every local authority in Great Britain. Gross expenditure includes all spending by local authorities for the provision of services which includes income raised through charges in providing those services. To calculate total local authority expenditure, we only included services that are consistently the responsibility of local authorities across all countries: CEP, social care, transport, central and those categorised as ‘other’ services, as defined in the revenue outturn guidance documents [2]. Data on local government expenditure and financing were sourced from the Place-Based Longitudinal Data Resource [33] (England), Scottish Local Government Finance Statistics [34] (Scotland) and StatsWales [35] (Wales); population estimates were sourced from the Office for National Statistics [36]. We investigated both variables of interest between 2009/10 and 2018/19 to cover the period of changes to local government resource allocation. Note that data available relates to financial years, i.e., from April 1st to March 31st, and for simplicity 2009 refers to financial year 2009/10.

The expenditure data is publicly available based on local authorities’ annual reports of expenditure in each of the three domains: cultural and related services, environmental and regulatory services, and planning and development services. Details on the individual services these include are available in the revenue outturn guidance documents [2]. Data were analysed at the aggregate level for CEP spending as a whole, as well as for the three domains described (in appendix 3), to investigate whether there are any variations in how services within CEP have been affected. There is a low level of missing data in the CEP expenditure dataset, with one of 3780 observations (LA-years) missing. Therefore, we concluded that excluding the missing observation from the analysis would not significantly affect results. We adjusted expenditure values for inflation, based on 2018 prices using the consumer price index (CPI) [37].

We investigated the changing patterns of expenditure by the following characteristics: nation, area deprivation, local government structure and rurality. We assigned local authorities to baseline within-country deprivation quintiles, based on the English Indices of Deprivation 2010 [38], the Welsh Index of Multiple Deprivation 2011 [39], and the Scottish Index of Multiple Deprivation 2012 [40]. We weighted IMD quintiles by population size such that each quintile contains the same number of people – this results in fewer local authorities in more deprived IMD quintiles as more deprived areas typically have larger populations. We also categorised local authorities into 5 groups based on their local government structure and country: English Shire Districts (two-tier), English Unitary Authorities (including Metropolitan Districts), London Boroughs, Scottish Local Authorities, and Welsh Local Authorities. These categories were used to visualise the differences between local authority structures. In further analysis quantifying the trends, we considered binary classifications of two-tier vs. unitary and London vs. outside of London, and a three-level categorical variable indicating country. We also classified areas as urban or rural using the Office for National Statistics’ mid-2018 estimates of population density for local authorities in the UK [41], and applying a binary classification of rurality using thresholds based on the Rural-Urban Classification [42], where the threshold for maximum population density in rural areas was 288 people per square kilometre. While population density is not strictly an urban-rural measure, it is a good proxy for which comparable data is available across Great Britain [43]. We present a map of rural and urban areas defined by population density in appendix 4.

### Analysis

First, we graphically assessed overall country-specific trends in CEP expenditure per capita and CEP as a share of local authority budgets between 2009 and 2018 by deprivation, rurality, and local government structure. We indexed values on the 2009 levels of spend to show the variations in trends over time. We also present graphs of the raw spending figures in appendix 5–10.

We then fitted a model to quantify trends in expenditure over time for all local authorities, stratified on the basis of our local characteristics of interest, including an assessment of country-specific time trends by deprivation and rurality, trends in unitary authorities compared to two-tier authorities, and trends in London boroughs compared to local authorities outside of London. This analysis however is complicated by the fact that individual area characteristics may be correlated with one another, e.g., urban areas are more likely to have higher levels of deprivation. For this purpose, we included all characteristics in a multivariable regression model. This enables us to understand the independent relationships between each local characteristic and the trend in CEP expenditure. For example, exploring the relationship of spending patterns and deprivation when we hold rurality constant. A generalised estimating equation (GEE) model was implemented for the outcome of annual gross CEP expenditure, using a log-link function and offset by the log-transformed population size of each local authority. Local authorities were weighted by their population size, to account for the variation in size of local authorities, such that our results show the per capita change in spend for local authorities when grouped by each characteristic, e.g. grouped by deprivation.

To understand how trends in the CEP share of total expenditure varied by these local characteristics we used a similar GEE model with the same outcome of annual gross CEP expenditure. Instead of using the log-transformed population size as the offset we use total local authority gross expenditure, such that the model output can be interpreted as a percentage of total local authority budgets spent on CEP services. We also weighted the analysis, this time by total local authority gross expenditure.

To assess country-specific time trends by deprivation and rurality for both expenditure per capita and CEP share of expenditure, we tested for interactions between time, country, and IMD or rurality as appropriate. We performed Chi-squared tests on nested models to determine whether these three-way interactions were statistically significant at the 5% level (see appendix 11). We included statistically significant three-way interactions in the final models.

To show the estimated trends in expenditure from these models, we present linear combinations of the relevant estimates for each characteristic (e.g. deprivation) stratified by country, whilst holding the other characteristics constant (e.g. rurality, local government structure). These can be interpreted as the trend in the specified sub-group, while the other characteristics are at their baseline level. For example, we present the estimate for annual percentage change in expenditure per capita for the least deprived local authorities in England, where rurality and local government structure are at their baseline levels (urban and two-tier respectively). In the results, we present the estimates for only the least and most deprived quintiles, to compare the trends at both extremes. Full results, including the intermediary quintiles, are given in appendix 12.

## Results

### Baseline spending patterns

Table 1 summarises characteristics of the dataset at the baseline year of 2009. In England, there was a clear gradient in the average CEP expenditure per capita, with more deprived local authorities spending more on CEP services. On the other hand, in Scotland and Wales, expenditure is similar across most quintiles, with only the most deprived quintile spending more on services. This gap in expenditure between the most deprived quintile and other local authorities is especially pronounced in Scotland.

In England and Scotland, urban local authorities spent an average of £60 and £75 more per capita on CEP services, respectively, compared to rural local authorities. However, in Wales, there does not appear to be a relationship between rurality and expenditure in 2009.

In England, 123 of the 324 local authorities are unitary, as opposed to being part of a two-tier structure. These unitary local authorities typically spent £79 more per capita on CEP services in 2009 than did the two-tier local authorities. Local authorities in London spent £105 more per capita than English local authorities outside of London. Overall, at baseline, local authorities in England and Wales spent similar amounts on CEP services; expenditure in Scotland was much higher.

**Table 1:** Descriptive statistics for local characteristics in 2009

	England		Scotland		Wales	
	Average CEP expenditure per capita (£)	Number of local authorities	Average CEP expenditure per capita (£)	Number of local authorities	Average CEP expenditure per capita (£)	Number of local authorities
IMD quintile						
Least deprived	311	89	492	9	348	6
2	332	78	483	6	374	5
3	378	66	474	7	364	3
4	396	50	473	5	374	4
Most deprived	491	41	684	5	431	4
Rural	335	99	483	19	379	10
Urban	395	225	558	13	374	12
Two-tier	334	201				
Unitary	413	123				
London	470	32				
Outside of London	365	292				
Total	381	324	521	32	376	22

### Trends in expenditure per capita

**Fig. 1:**
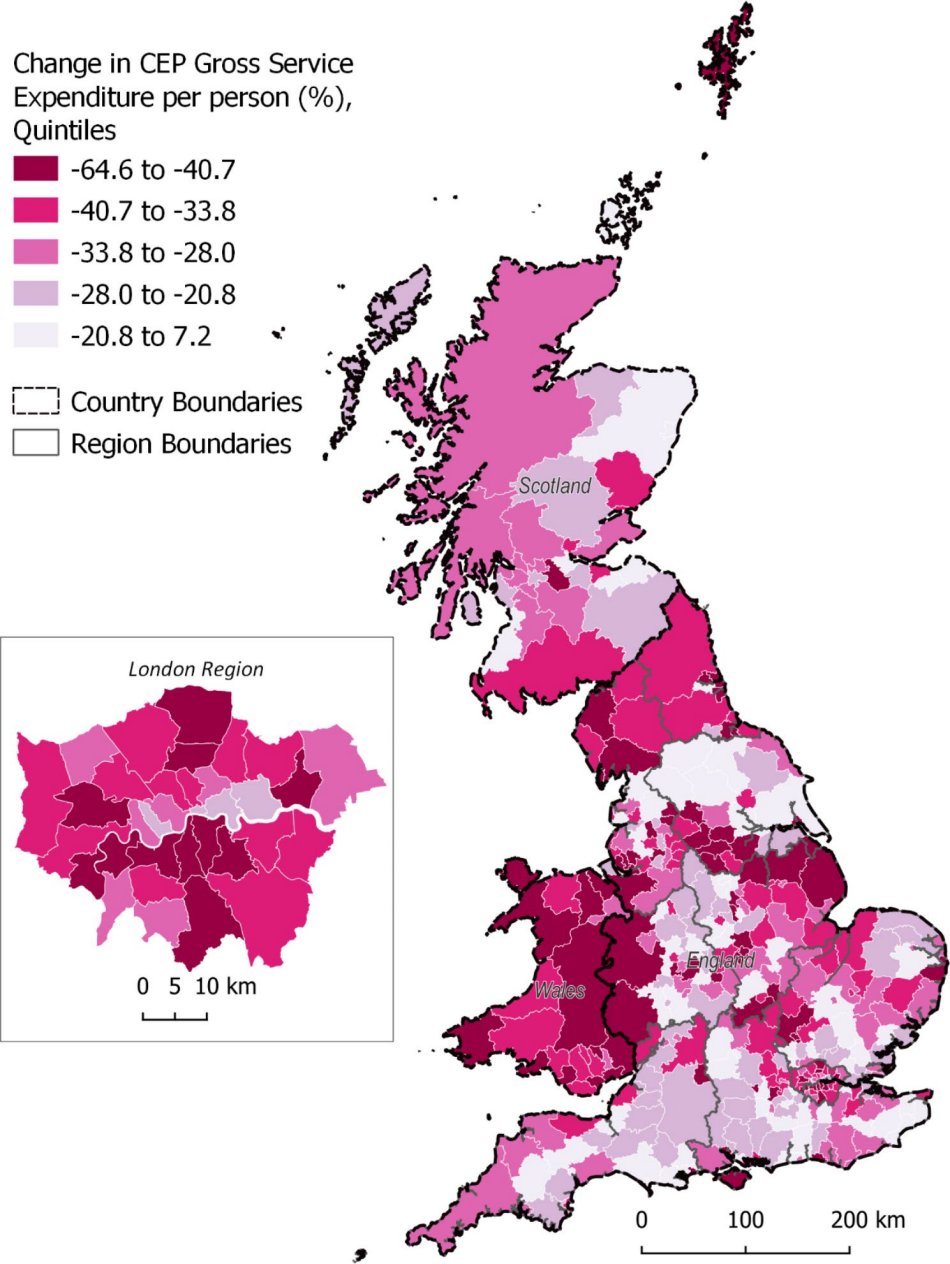
Percentage change in CEP gross expenditure per capita between 2009 and 2018, by lower-tier local authorities

There is large variation in spending patterns on CEP services between local authorities in Great Britain. Figure 1 illustrates the percentage change in CEP gross expenditure per capita between 2009 and 2018, for lower-tier local authorities in Great Britain. Local authorities with the largest reductions in expenditure are primarily found in Wales and parts of England, such as the North and London boroughs. Appendix 13 provides a population-weighted cartogram of the same changes in CEP spend.

**Fig. 2:**
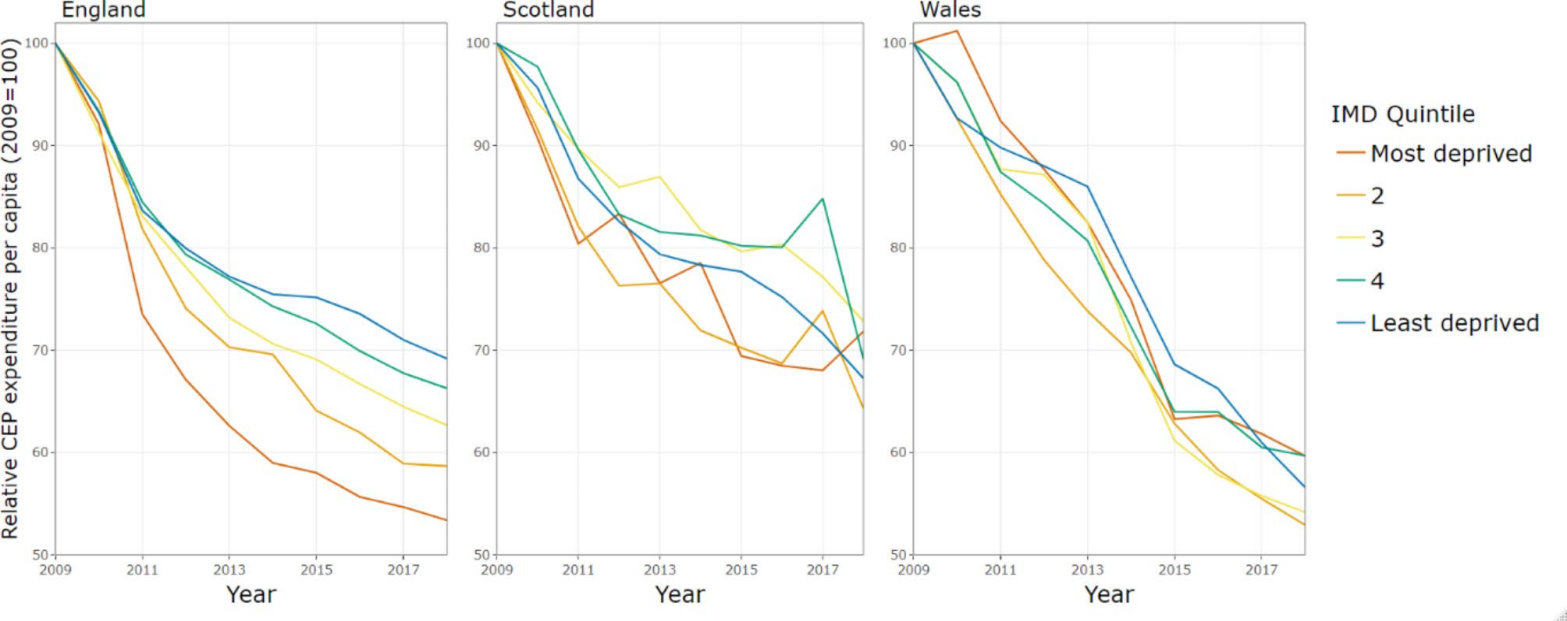
Gross expenditure per capita on CEP services by country and level of deprivation, indexed on 2009 spending

The gross expenditure per capita on CEP services between 2009 and 2018, stratified by within-country IMD quintiles is shown in Fig. 2. In England, there was a much steeper decline in expenditure in more deprived local authorities relative to the least deprived local authorities. In contrast, in Scotland and Wales the decline follows a similar trend across deprivation quintiles.

**Fig. 3:**
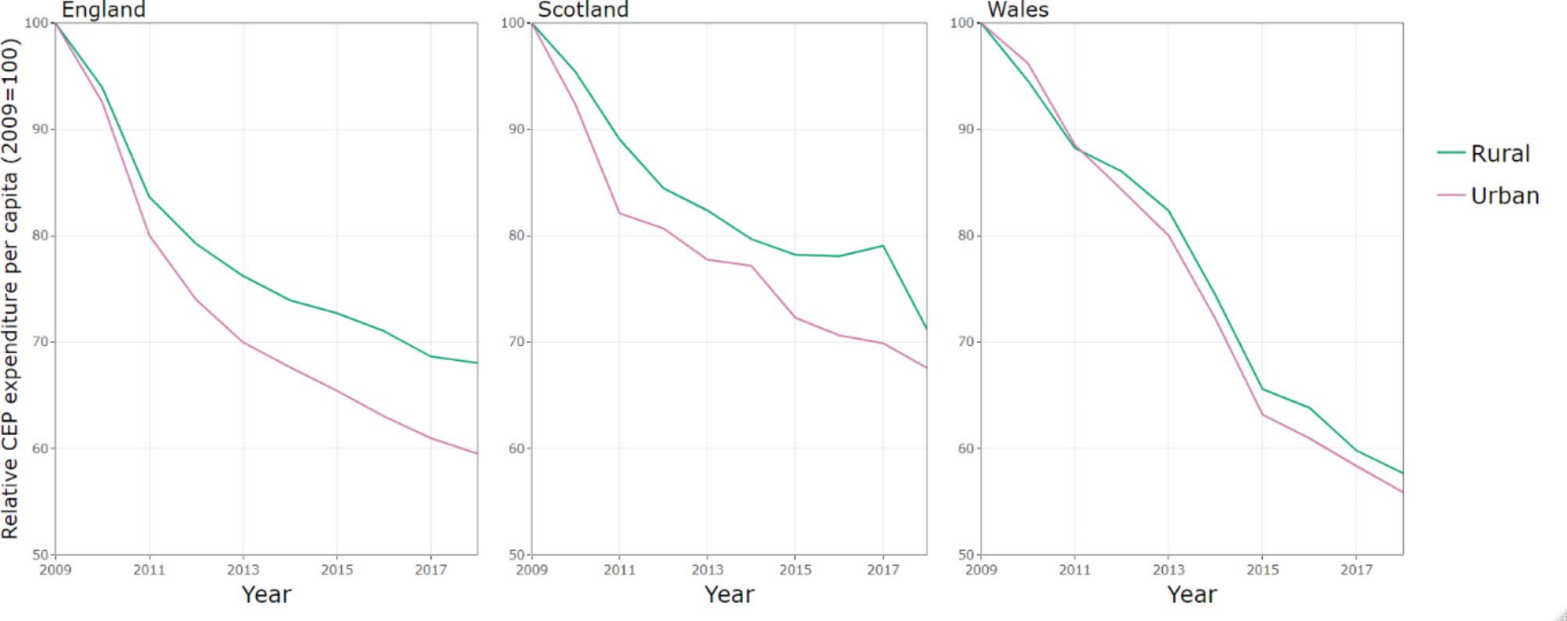
Gross expenditure per capita on CEP services by country and rurality, indexed on 2009 spending

Figure 3 shows per capita CEP spend stratified by rural and urban for the three countries. In England and Scotland, the decline in expenditure has been steeper in urban areas (which started with higher baseline levels of spend), whilst the decline in Wales has been similar across urban and rural local authorities.

**Fig. 4:**
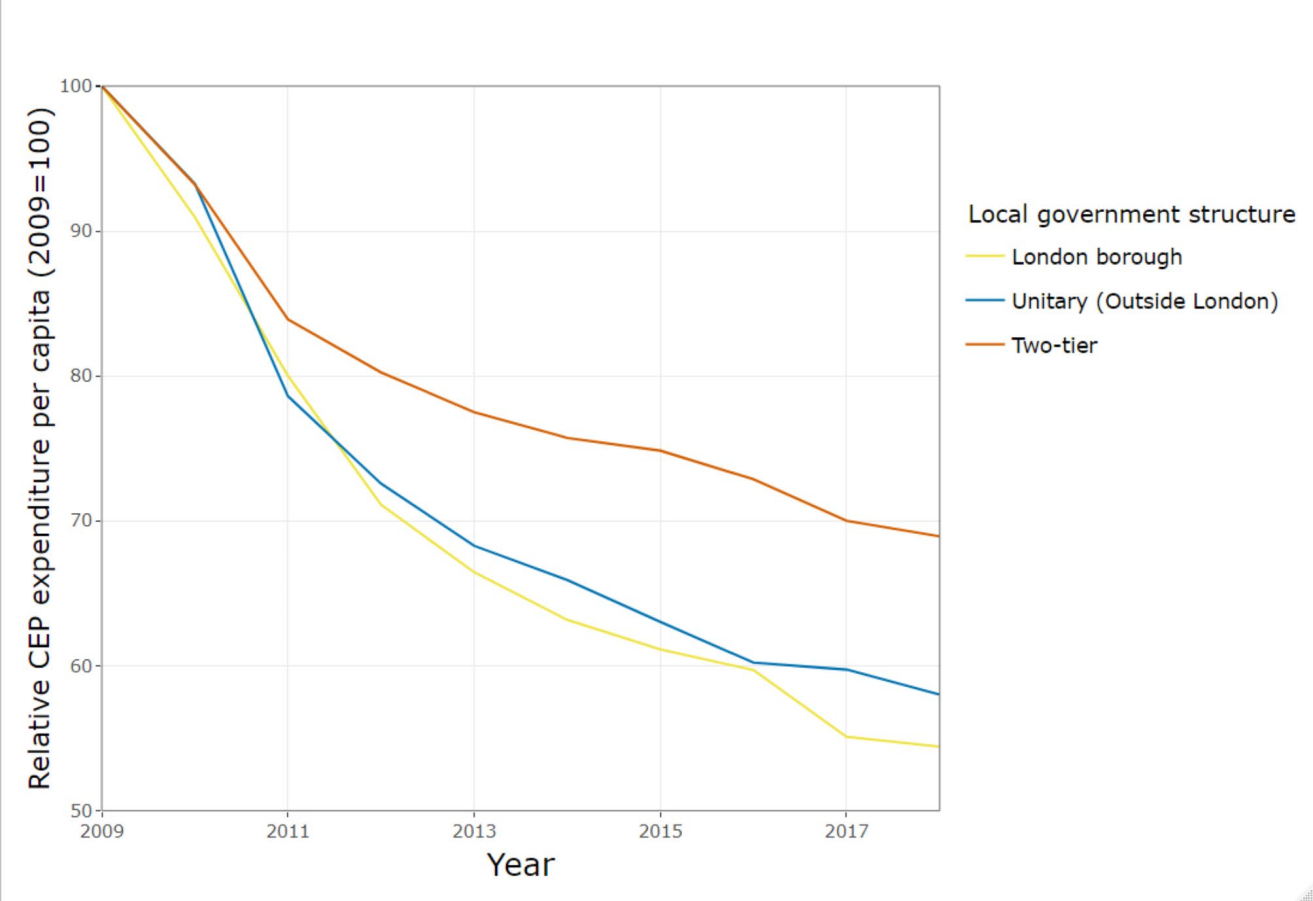
Gross expenditure per capita on CEP services by local government structure in England, indexed on 2009 spending

Figure 4 shows per capita CEP expenditure between 2009 and 2018, stratified by local authority structure in England. This shows that whilst CEP spend in London metropolitan boroughs was highest at baseline it reduced the most over this time frame, compared to other local authority structures. Unitary authorities outside London started with higher expenditure than two-tier areas (Shire districts) but reductions are greater such that by 2018 there is no difference in CEP expenditure between these local authority types.

Table 2 provides the rates of decline estimated by the generalised estimating equation (GEE) models for CEP expenditure per capita, indicating the independent effect of each characteristic (i.e. the effect when holding the other characteristics constant). We found that trends on the basis of rurality and deprivation varied by country (i.e. 3-way interactions were statistically significant, see appendix 11 for further details). We show these results stratified by country. For simplicity we present results only for the most and least deprived quintiles of LAs here, although results for all IMD quintiles are given in appendix 12.

In England, the annual reduction in budget was greatest for local authorities in the most deprived quintile, at -7.46% (95% CI: -8.86, -6.03) per year, compared to -4.45% (95% CI: -5.60, -3.29) per year in the least deprived quintile. However, differential trends in expenditure by level of deprivation were not evident in Scotland and Wales. For example, in Wales, the least and most deprived local authorities have experienced similar levels of budget cuts at -5.77% (95% CI: -6.50, -5.04) per year and − 6.07% (95% CI: -6.78, -5.36) per year, respectively [Table 2].

Differential trends in expenditure are also apparent across other local authority characteristics even when accounting for deprivation. In Scotland, local authorities in urban areas have experienced larger budget cuts than those in rural areas, at -5.03% (95% CI: -5.89, -4.17) annually compared to -3.86% (95% CI: -5.08, -2.63) per year [Table 2]. By contrast, in England, once we account for trends by level of deprivation and local government structure, there was no relationship between declines in expenditure and whether the local authority was in a rural or urban area. In Wales, as we saw in the descriptive analysis (Fig. 4), local authorities have experienced similar budget cuts across rural and urban areas, even when accounting for deprivation.

In England, when adjusting for deprivation and rurality, we found that local government structure has modified the trend in CEP expenditure over this time period. Unitary local authorities in England outside London have been more substantially affected. When adjusting for deprivation and rurality, annual change in expenditure per capita for these unitary authorities has been − 4.45% (95% CI: -5.60, -3.29) per year whilst those in two-tier areas have seen lower reductions, at -3.12% (95% CI: -4.19, -2.04) annually.

**Table 2:** Results of generalised estimating equations modelling longitudinal CEP expenditure by local characteristics

Characteristic	Annual percentage change in CEP expenditure per capita (95% CI)
**England**	
Least deprived IMD quintile	-4.45 (-5.60, -3.29)
Most deprived IMD quintile	-7.46 (-8.86, -6.03)
**Scotland**	
Least deprived IMD quintile	-5.03 (-5.89, -4.17)
Most deprived IMD quintile	-3.98 (-5.88, -2.05)
**Wales**	
Least deprived IMD quintile	-5.77 (-6.50, -5.04)
Most deprived IMD quintile	-6.07 (-6.78, -5.36)
**England**	
Rural	-4.86 (-5.67, -4.05)
Urban	-4.45 (-5.60, -3.29)
**Scotland**	
Rural	-3.86 (-5.08, -2.63)
Urban	-5.03 (-5.89, -4.17)
**Wales**	
Rural	-5.72 (-6.90, -4.54)
Urban	-5.77 (-6.50, -5.04)
**Local government structure (England LAs only)**	
Two-tier (Shire Districts)	-3.12 (-4.19, -2.04)
Single-tier councils outside London (unitary authorities and metropolitan districts)	-4.45 (-5.60, -3.29)
London boroughs	-5.10 (-6.44, -3.73)

Rates presented are the linear combinations of relevant estimates for each local characteristic considered, holding all other characteristics constant at their base level

### Trends in CEP share of expenditure

**Fig. 5:**
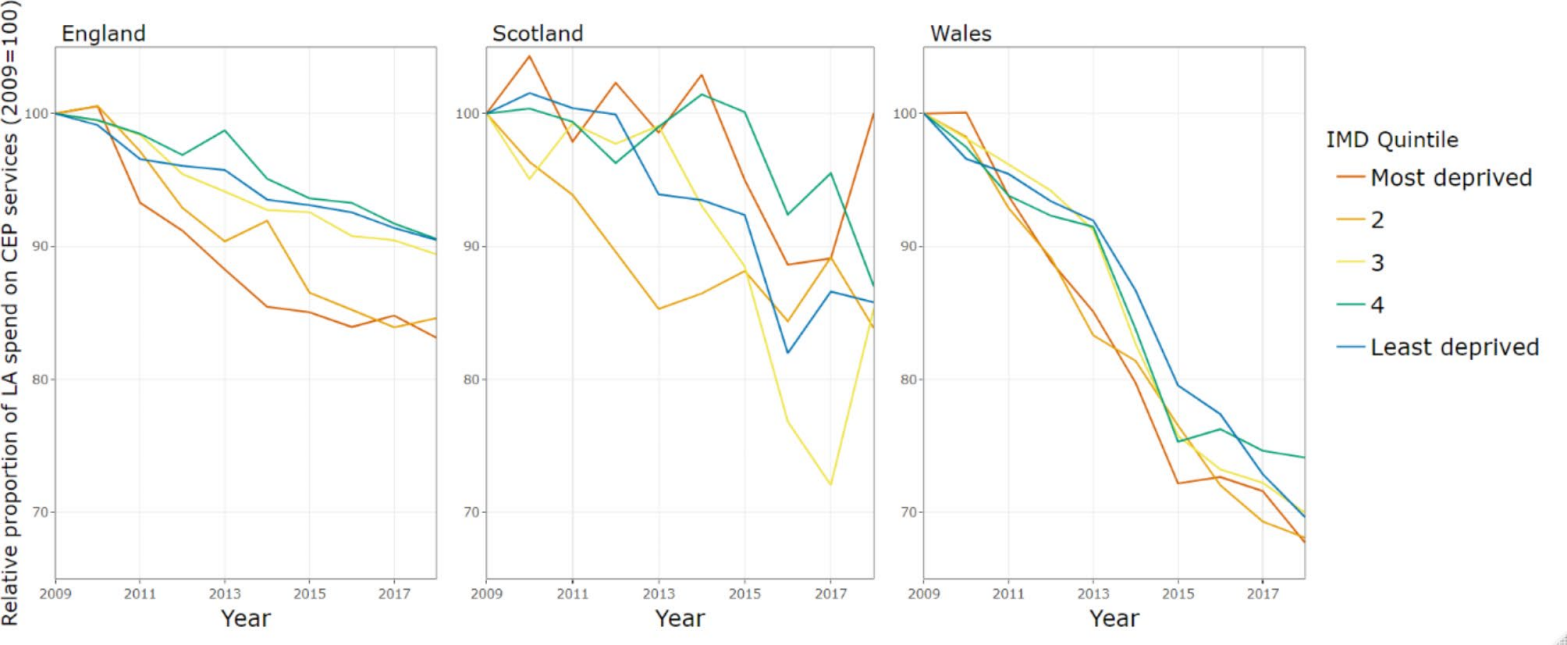
Proportion of gross expenditure on CEP services by country and level of deprivation, indexed on 2009 spending

Figure 5 illustrates CEP expenditure over time, as a proportion of total local authority expenditure, between 2009 and 2018, stratified by within-country IMD quintiles. Overall, local authorities in Wales have reduced their CEP share of budgets to a greater extent than England and Scotland. In England, there has been a slightly greater decline in share of total expenditure on CEP in more deprived local authorities, whilst in Scotland the pattern is more mixed, CEP share has been maintained at a similar level in the most deprived areas but has decreased in the least deprived areas. In Wales there has been a similar decline across all deprivation quintiles.

**Fig. 6:**
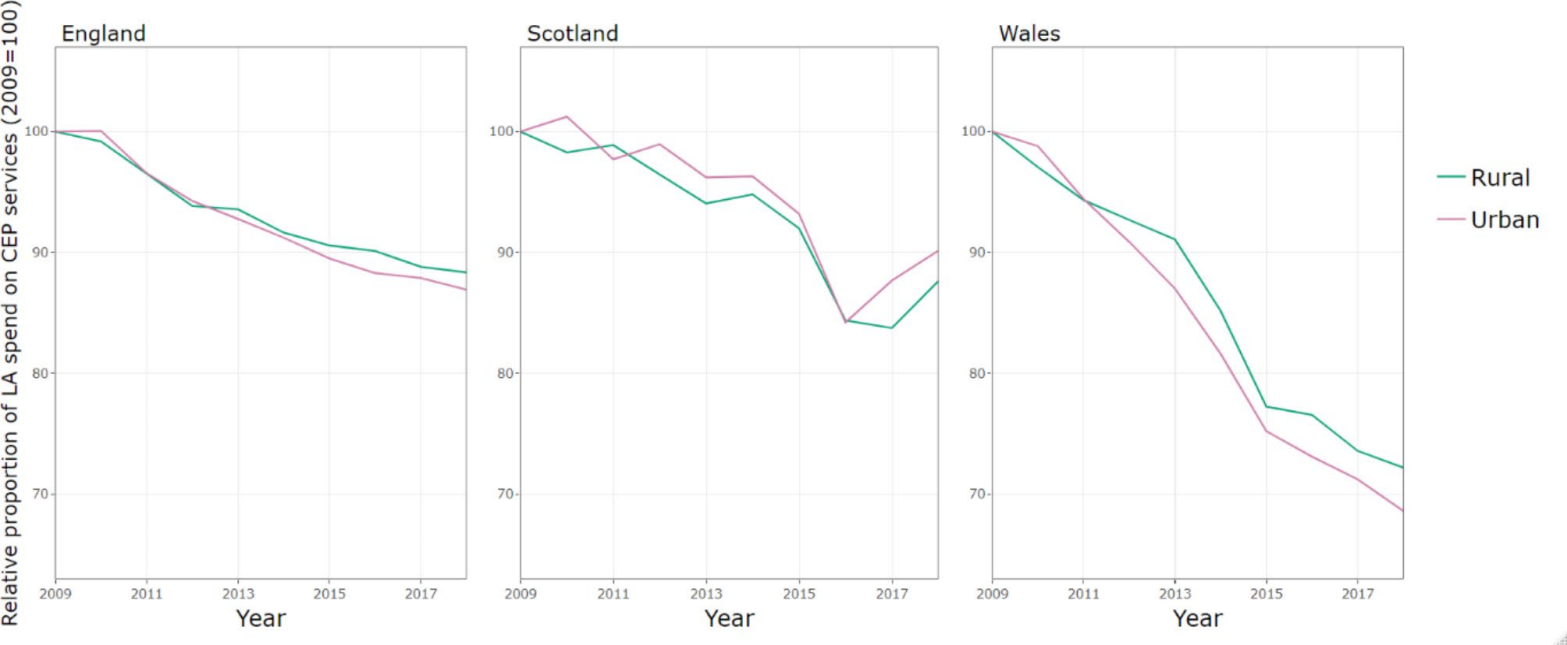
Proportion of gross expenditure on CEP services by country and rurality, indexed on 2009 spending

Figure 6 shows the CEP share of expenditure each year stratified by rural and urban for the three countries. In England rural areas spend a greater share of their budgets on CEP services, whilst in Scotland the CEP share in urban areas is greater. In Wales the CEP shares are very similar across rural and urban areas. Whilst all countries reduced their share of spending on CEP, the rural-urban pattern remains fairly similar over time.

**Fig. 7:**
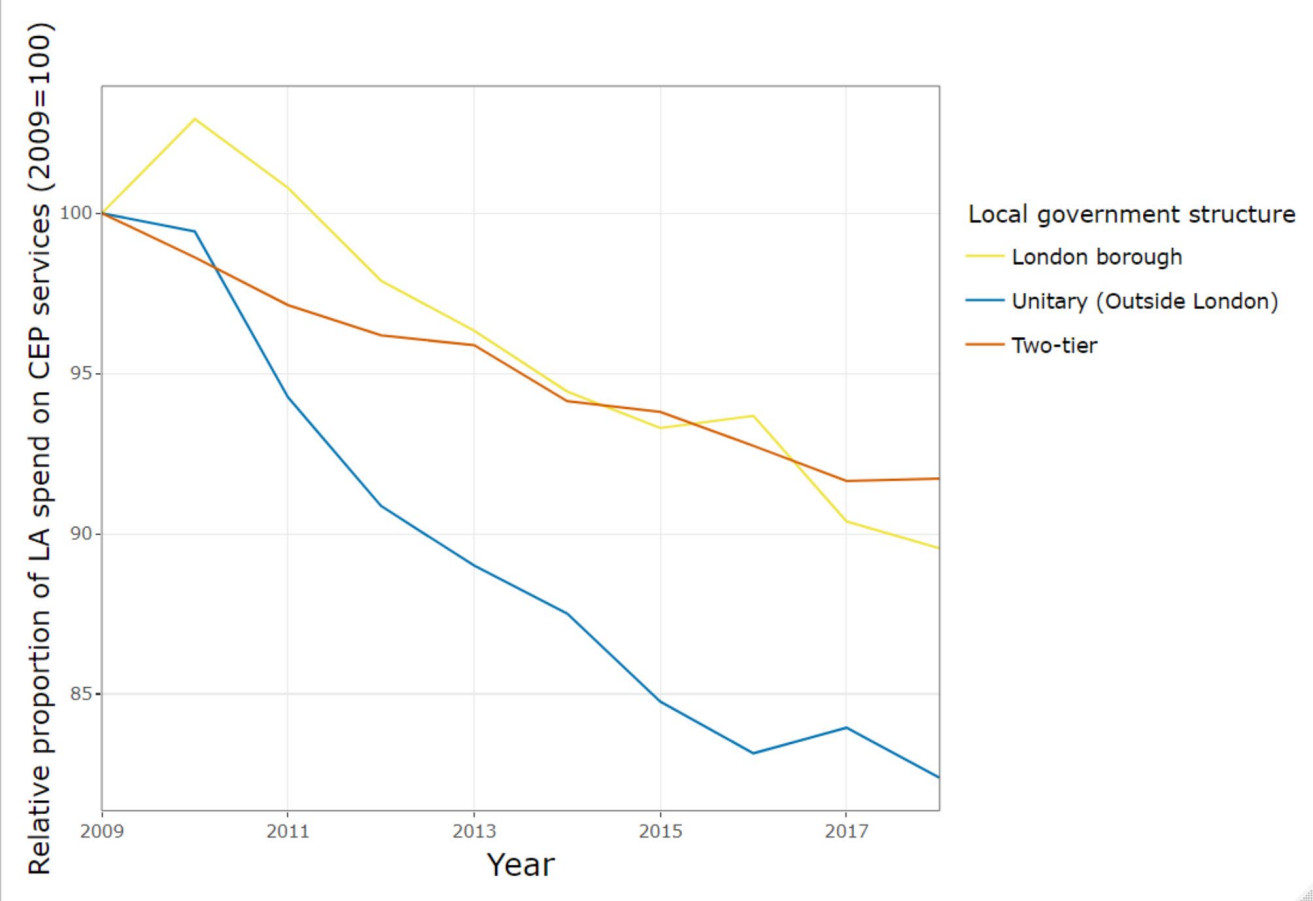
Proportion of gross expenditure on CEP services, by local government structure in England, indexed on 2009 spending

Figure 7 illustrates the trend in CEP expenditure as a proportion of total local authority expenditure, for different local government structures in England. Unitary authorities outside London have reduced their CEP share of budget from 24.1% to 2009 to 19.9% in 2018. This cut is larger than two-tier areas which reduced their CEP share from 23.4% to 2009 to 21.4% in 2018. Similarly, London borough local authorities have experienced only a small reduction in CEP share of budgets, from 20.5% to 2009 to 18.3% in 2018.

Table 3 shows the relative change in the share of CEP spend as a proportion of total local authority expenditure estimated by the GEE models, indicating the independent effect of each characteristic on this measure. As with per capita spend, we found that trends on the basis of rurality and deprivation varied by country, and so we show these results stratified by country (see appendix 11 for further details). Full results for all IMD quintiles are given in appendix 12.

In England, after accounting for rurality and local government structure, the share of total expenditure on CEP has decreased to a greater extent in the most deprived areas, by -3.14% (95% CI: -4.03, -2.24) annually compared to -1.37% (95% CI: -2.16, -0.57) per year in the least deprived quintile [Table 2]. In Scotland, the CEP share has stayed fairly constant in the most deprived areas (0.21%, 95% CI: -0.99, 1.42 annually), whilst declining in the least deprived areas. In Wales, both least and most deprived have decreased the CEP share substantially, by -3.95% (95% CI: -5.01, -2.87) per year and − 4.86% (95% CI: -5.70, -4.02) per year, respectively. In England, holding deprivation constant, we see a greater decline in the share of total funding on CEP services in rural areas at -2.30% (95% CI: -2.92, -1.68) per year, compared to urban areas at -1.37% (95% CI: -2.16, -0.57) per year. In Scotland and Wales, the cuts are similar in both urban and rural areas.

In England, there are differences related to local government structure. The CEP share has declined more in unitary authorities outside London, with an annual change in CEP share of -1.37% (95% CI: -2.16, -0.57) per year compared to -0.20% (95% CI: -0.89, 0.50) per year in local authorities within two-tier systems. In London, after accounting for trends by deprivation, the share of total budgets spent on CEP changed by -0.40% (95% CI: -1.34, 0.54) annually.

**Table 3:** Results of generalised estimating equations modelling longitudinal CEP expenditure by local characteristics

Characteristic	Annual percentage change in CEP share of expenditure (95% CI)
**England**	
Least deprived IMD quintile	-1.37 (-2.16, -0.57)
Most deprived IMD quintile	-3.14 (-4.03, -2.24)
**Scotland**	
Least deprived IMD quintile	-2.96 (-4.19, -1.71)
Most deprived IMD quintile	0.21 (-0.99, 1.42)
**Wales**	
Least deprived IMD quintile	-3.95 (-5.01, -2.87)
Most deprived IMD quintile	-4.86 (-5.70, -4.02)
**England**	
Rural	-2.30 (-2.92, -1.68)
Urban	-1.37 (-2.16, − 0.57)
**Scotland**	
Rural	-2.50 (-3.85, -1.14)
Urban	-2.96 (-4.19, -1.71)
**Wales**	
Rural	-3.52 (-4.41, -2.63)
Urban	-3.95 (-5.01, -2.87)
**Local government structure (England LAs only)**	
Two-tier (Shire Districts)	− 0.20 (-0.89, 0.50)
Single-tier councils outside London (unitary authorities and metropolitan districts)	-1.37 (-2.16, − 0.57)
London boroughs	− 0.40 (-1.34, 0.54)

Rates presented are the linear combinations of relevant estimates for each local characteristic considered, holding all other characteristics constant at their base level

## Discussion

### Summary of findings

Cultural, environmental, and planning services have an important impact on population health through a variety of pathways, yet under the past decade of austerity, cuts to local government budgets have dramatically reduced investment in these services. In this study, we investigated trends in local authority CEP expenditure across Great Britain since austerity measures were implemented in 2010 and considered how these trends may vary according to characteristics of local authorities. Both in absolute terms and relative to the total spending on all services, local authorities have substantially reduced spending on CEP services since 2010. In England, more deprived areas experienced the deepest cuts, in both absolute and relative terms. In Wales, cuts to CEP as a proportion of spending on all services were particularly severe. Local authorities with a unitary local government structure have experienced more substantial cuts than those with a two-tier structure. Patterns by rurality were more complex and varied depending on the country.

### Results in the context of past research

Previous research investigating the changes in local government funding since the implementation of austerity has focussed on local government services as a whole [44–47, 49]. In our study, we focussed on CEP services, enabling us to identify additional trends specific to these services. These services are of particular interest as, despite their role in promoting health [13, 15, 25], for the most part they are not statutory and so might be deprioritised disproportionately when budgets are constrained. Within the service lines of CEP expenditure, cultural and planning services are especially at risk of disproportionate budget cuts due to their discretionary nature, as compared to environmental services which are largely statutory (see appendix 3).

Previous research [46] suggests that more deprived areas have been impacted more by cuts to central government funding as they are more reliant on these grants, due to a lower capacity to raise funding through taxation of local properties and businesses. Therefore, cuts to central government grants have directly led to cuts in local government spending in these areas. Hastings et al. report that the most deprived areas in England experienced percentage cuts in spending per capita 4.3 times higher than the least deprived areas between 2010 and 2014 [46]. Our study shows budget cuts have especially impacted CEP services in deprived areas of England, and descriptive analysis of the income generated from CEP services suggests less deprived areas have had a greater capacity to maintain this income (see appendix 14). This deprivation trend in budget cuts is not apparent in Scotland and Wales. Reduced budgets may be due to a larger proportion of vulnerable people in deprived areas, requiring statutory services such as social care to be prioritised, in addition to a lower capacity to maintain income from service provision.

Similarly, budget cuts have been unequally distributed across rural and urban areas. As Gray and Barford describe, rural areas are less reliant on central government grants as they are more able to raise funding through local taxation [44]. Therefore, cuts to central government grants have had less impact on total local authority budgets in rural areas as compared with towns and cities. Our research shows that, despite this, rural areas in England seem to be deprioritising CEP services. This may be due to the differing demographics of rural areas, such as an older population requiring more social care support.

Additionally, previous evidence of a relationship between the structure of local governments and the size of budget reductions experienced supports our findings. Local governments in two-tier systems, such as those in counties, have seen smaller cuts to their overall budgets. For example, the National Audit Office (NAO) report showed that metropolitan districts’ (unitary) overall budgets reduced by 41% between 2010 and 2015. In comparison, county councils (two-tier) experienced a 33% reduction. This is likely due to metropolitan districts having less capacity to raise revenue: on average only 33.7% of their income is derived from council tax, compared to 56.4% in county councils [45]. This pattern is likely exaggerated in our study of CEP services, as the services are largely discretionary and so more likely to be cut by unitary authorities with responsibilities over important statutory services, such as social care.

Our study shows country-level differences in the effects of austerity on local governments within Great Britain. Scotland, Wales, and Northern Ireland are devolved nations, meaning that they have different resource allocation mechanisms and autonomy over some systems including local government. Between 2009/10 and 2016/17, average total local government funding reduced by 12.1% and 11.5% in Wales and Scotland, respectively [44]. Although these cuts are significant, they are smaller than that experienced by local governments in England, at an average of 23.7%, suggesting that devolution of nations potentially offers local governments some protection from overall budget cuts. This aligns with the recent UN report detailing the austerity mitigation efforts implemented by the devolved nations [48]. However, our study found this protective effect of devolution did not extend to CEP services, with Wales experiencing the largest per capita budget cuts for CEP services, followed by Scotland and then England. This highlights the different responses countries had to the overall budget cuts, with the Scottish and Welsh local authorities deprioritising CEP services to a greater extent than English local authorities. While devolution might not have protected Scottish and Welsh local authorities against total CEP cuts, it has allowed the countries some discretion over how to distribute those cuts. This has resulted in a more equitable distribution of budget cuts in the devolved nations than was evident in England.

### Strengths and limitations

The main strength of our study is the use of longitudinal data. This enabled us to consider expenditure patterns over time, as opposed to differences between local authorities at a fixed time point that cross-sectional data would illustrate. The data used is of high quality as a result of cleaning and collation by the Place-based Longitudinal Data Resource [33].

Additionally, this is the first study of analysis of trends in expenditure on CEP services in Great Britain, providing vital evidence on the resources allocated to these important health-promoting services. Prior research has investigated expenditure trends for total local authority expenditure, with only one paper analysing cultural expenditure trends limited to England [49]. Assessing the trends in both CEP expenditure per capita and CEP expenditure as a share of total local authority budgets allowed us to explore the extent to which changes are due to cuts in total local authority budgets or changes in prioritisation of CEP services.

Finally, a strength of our study is that we have considered local action on health and health inequalities within the context of national policy on local government funding. Previously, place-based approaches have been criticised for focusing exclusively on local factors while ignoring national policy and other macro-level structural determinants of inequality [50]. It’s important that, in addition to more equitable place-based investment strategies, broader policy actions on the upstream determinants of health inequalities are taken to address the widening inequalities in the UK [5].

One limitation of our study is the time period considered. It might be beneficial to analyse a longer period, to compare the trends of expenditure before and after austerity was introduced. Unfortunately, no comparable data is available before 2007 so there are not enough data points before the introduction of austerity measures to provide meaningful comparisons.

Our study was also limited by the availability of comparable data in Northern Ireland. Including analysis of trends in expenditure in Northern Ireland could have strengthened our conclusions on the influence of devolution.

The use of broad categories of spend in our analysis might also be considered a limitation, as some services included may have weaker associations with health outcomes than others. However, in using these categories rather than more granular data on specific services, we account for potential differences in categorisation between local authorities and over time.

A further limitation of our study is the use of a binary measure of population density as a proxy for rurality. More complex measures might consider population size, how built up or sparse an area is, or proximity to urban areas. However, population density is the most consistently reported measure across Great Britain and is considered a good proxy for rurality [43].

Finally, our study is limited by the use of gross spending data, which is total spend on service provision including income generated from service use. This complicates the interpretation of trends in terms of service provision, as increased income through fees and charges may come at the expense of affordability for some groups. So, a lower level of cuts in some local authorities due to increased income generation through fees and charges may mask potential adverse trends, whereby inequalities in use of CEP services within local authorities could increase as CEP services (e.g., leisure facilities) become less affordable. Unfortunately, consistent data on the income generated from CEP services was not available for Wales. In appendix 14, we investigated the trends in CEP service income in England and Scotland and found that income also fell over this time and fell to a greater extent in more deprived areas, i.e., following a similar pattern as trends in net expenditure. This suggests more affluent areas have had a greater capacity to maintain this income.

### Conclusions and implications for policy and research

Whilst place-centred investment in the social determinants of health is widely recognised as a strategy for improving population health and health equity, the UK has witnessed a steady erosion in such investment. This erosion is directly related to cuts in the funding of local authorities by central government, but also appears to reflect decisions, taken at local level, to invest a smaller proportion of the available budgets on CEP services. This suggests a gradual abandonment of place-centred policies at local level, particularly in areas with higher need. The reasons why CEP services are becoming less of a local priority are not clear and should be assessed as a matter of urgency, as should the impacts of these budgeting decisions on health and health equity. More investment and more equitable investment in these services is needed as a route to improving health and regional prosperity; this should be a key priority of the UK government’s ‘levelling up’ agenda as we emerge from the COVID-19 pandemic [51].

## Electronic supplementary material

Below is the link to the electronic supplementary material.

Supplementary Material 1

## Data Availability

The datasets used and/or analysed during the current study are available from https://pldr.org/ and further guidance for the data can be forwarded to the corresponding author on reasonable request.

## List of abbreviations

CEP: Cultural, environmental and planning
LA: Local authority
UK: United Kingdom
IMD: Index of Multiple Deprivation
GEE: Generalised estimating equation
NAO: National Audit Office
CPI: Consumer Price Index
