# Supplementary material for: Inequalities in local government spending on cultural, environmental and planning services: a time-trend analysis in England, Scotland, and Wales
Source: BMC Public Health. 2023 Feb 28;23:408. doi: 10.1186/s12889-023-15179-9 (PMC9974056; doi:10.1186/s12889-023-15179-9)

## Appendix 1

The four main sources of funding for local authorities are: central government grants; fees and service charges; business rates, a local property tax on businesses; and council tax, a local property tax on households. Central government grants are allocated to each country using the Barnett formula, which allocates funding annually based on the overall change in UK funding, and the population size and level of devolution in the country(Great Britain & Treasury, 2020; Keep, 2020). There are some differences in allocation between countries, for example, Wales’ Barnett formula incorporates a needs-based aspect to protect funding from falling below a certain level. This aspect is not present in Scotland’s formula, and Scotland has increasing autonomy over funding, such as greater devolution of tax and spending powers. Across all countries, central government grants account for the largest proportion of local authority funding: 50% in England’s 2018-19 budget(Ministry of Housing, Communities & Local Government, 2018). The amount of grant each council receives is determined by their country’s central government, though all countries employ a similar method of assessing relative needs and resources of councils. This assessment is made using formulas that account for indicators of need, such as local demographics, and indicators of resource, such as the council tax base of the local authority, in comparison with others in the country(Finnigan, 2018; Thomas, 2018).

## Appendix 2

For England, data on CEP services spending of individual LAs were compiled using the General Fund Revenue Account Outturn - Revenue Outturn Service Expenditure Summary (RSX) tables, published annually by the UK Department for Communities and Local Government.

To provide a consistent time series of allocations of service expenditure across LAs, figures from all the different types of LAs were mapped to lower-tier LAs. Local government structure in some areas of England is hierarchical, with two main tiers of local government, upper and lower, that are geographically nested. In addition, there are several other types of LAs, such as police, transport and combined authorities, each with its own area and domain of activity. The allocation of funds within these is similarly reported at multiple levels.

In order to make comparisons possible, all figures from upper-tier authorities (i.e. County Councils) were disaggregated to lower-tier level (i.e. Shire Districts) using look-up tables provided by the Office for National Statistics (ONS). Where local government organisations spanned more than one upper-tier LA, we apportioned the total allocation to each LA based on their annual population estimates, as provided by ONS. In particular, these LA types are:

- The Greater London Authority
- Combined Authorities
- Police and Crime Commissioner and Chief Constable Authorities
- Fire and Rescue Authorities
- Waste Authorities
- Transport Authorities

All income figures for the above types of authorities have been included in our calculations and compiled into annual figures, with the exception of National Park Authorities; however, these are a small number of authorities established to maintain National Parks, with relatively low levels of funding and spending.

For Wales, data on CEP services spending of individual LAs were compiled using the Revenue Outturn Expenditure tables, published annually by the Welsh Government StatsWales. Data on service income is not published. All local authorities in Wales are unitary, and their geography has remained consistent over the time period considered.

For Scotland, data on CEP services spending and income of individual LAs were compiled using the Scottish Local Government Finance Statistics tables, published annually by the Scottish Government’s Local Government and Communities Directorate. All local authorities in Scotland are unitary, and their geography has remained consistent over the time period considered.

Across all countries, figures are given on the basis of financial years, i.e., from April 1st to March 31st, and for the analysis of annual trends, the first calendar year was used as reference.

## Appendix 3

Proportion of gross CEP expenditure spent on individual service lines, in England, Scotland and Wales in 2009 and 2018.


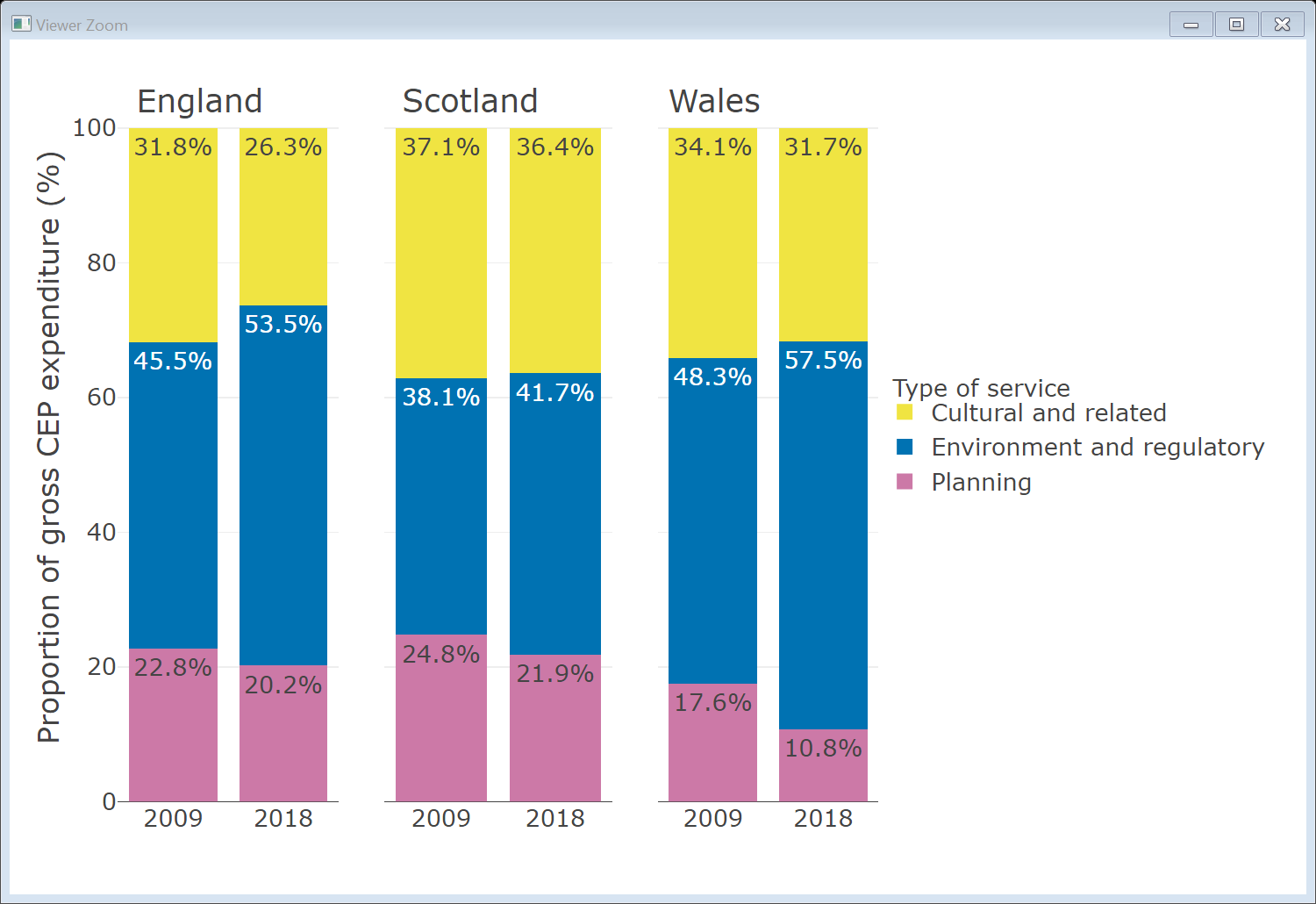


## Appendix 4

Rural and urban areas of Great Britain, as defined by population density of above or below 288 persons per km^2^.

##
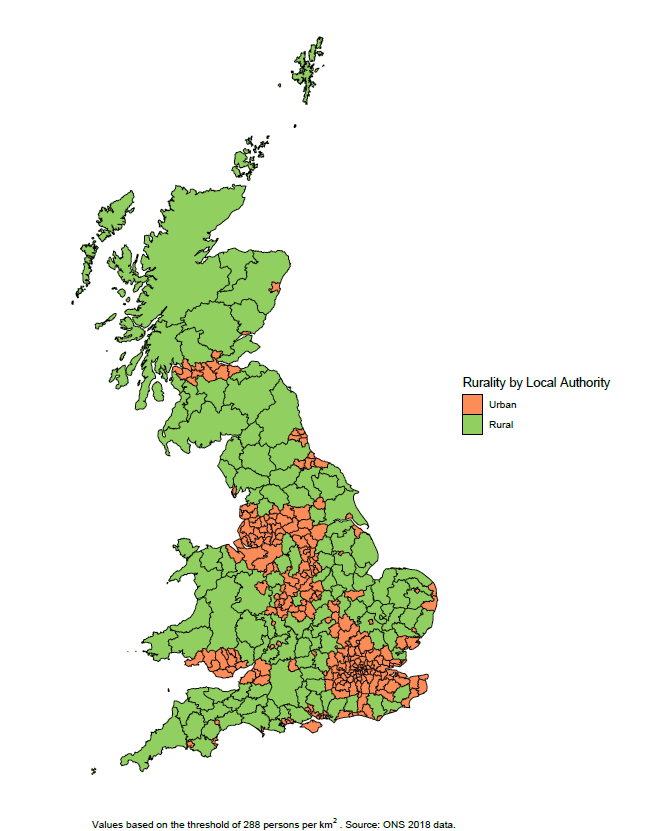
Appendix 5

Gross expenditure per capita on CEP services by country and level of deprivation

##
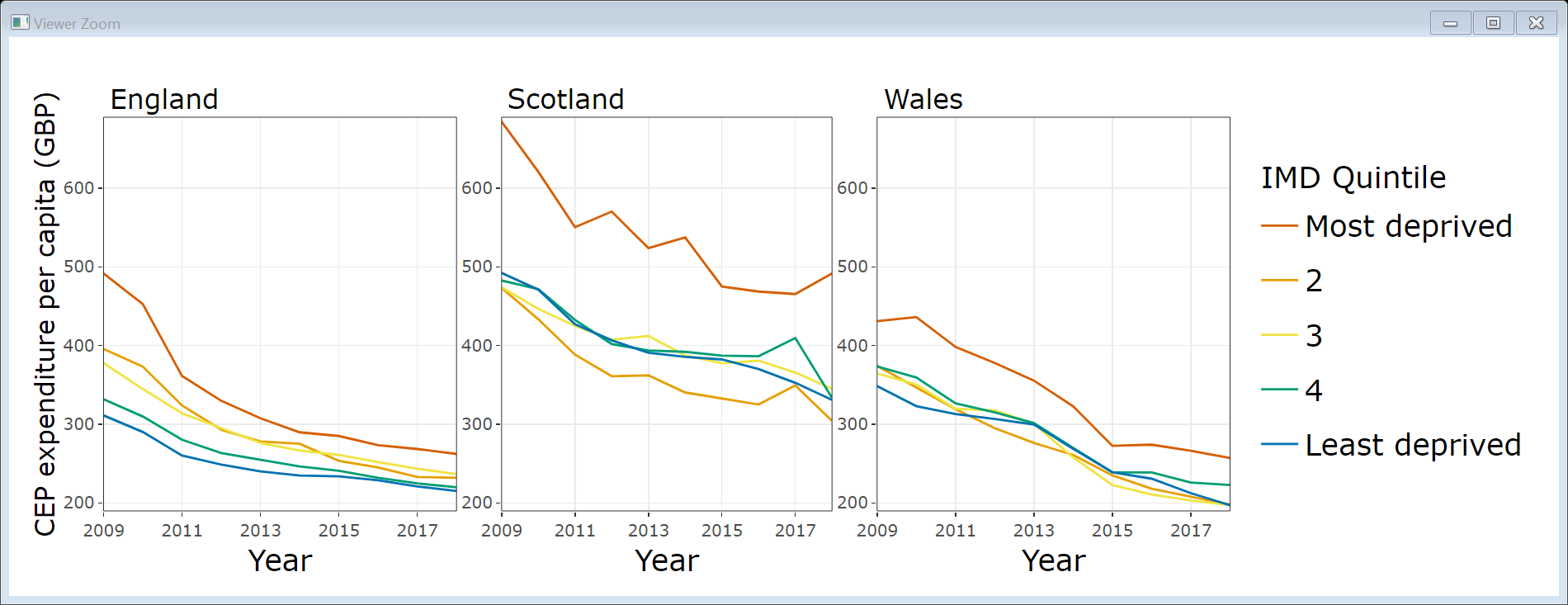


## Appendix 6

Gross expenditure per capita on CEP services by country and rurality

**
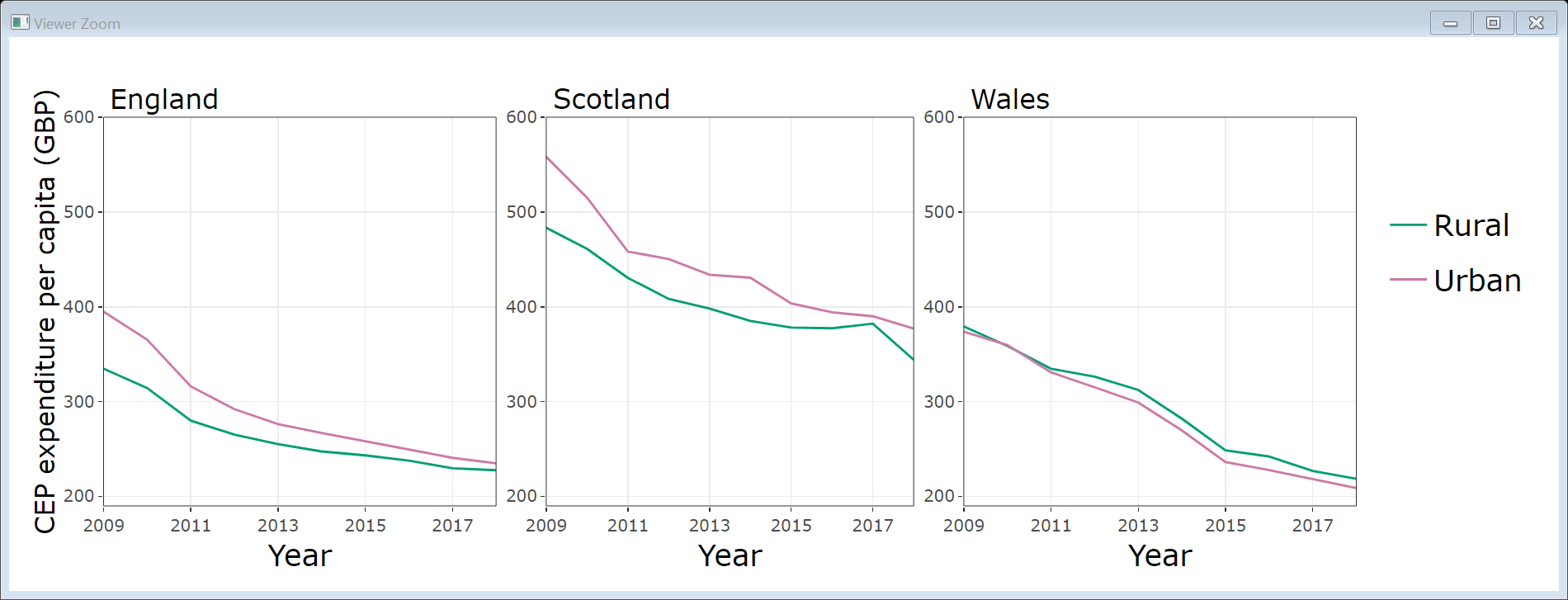
**

## Appendix 7

Gross expenditure per capita on CEP services by local government structure in England.
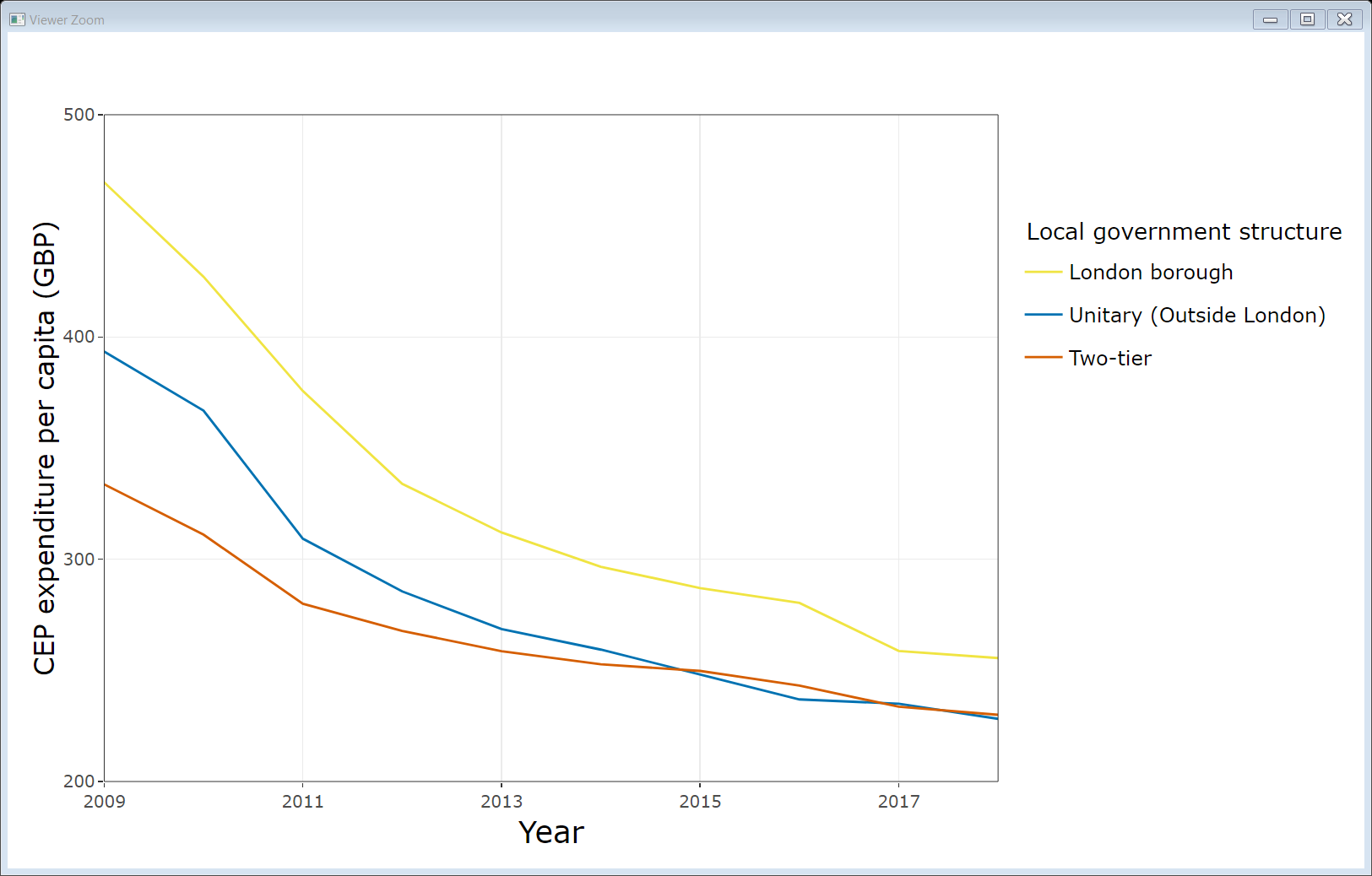


## Appendix 8

Proportion of gross expenditure on CEP services by country and level of deprivation


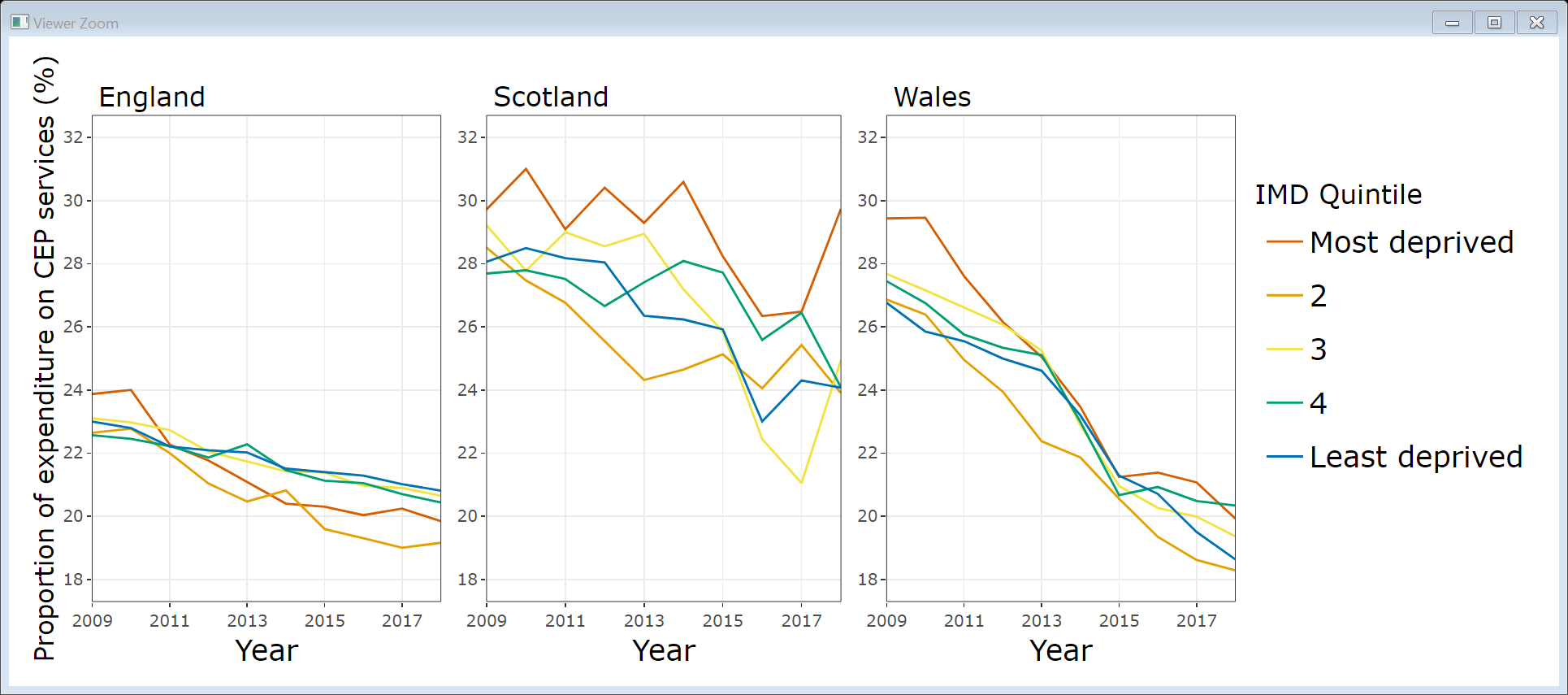


## Appendix 9

Proportion of gross expenditure on CEP services by country and rurality


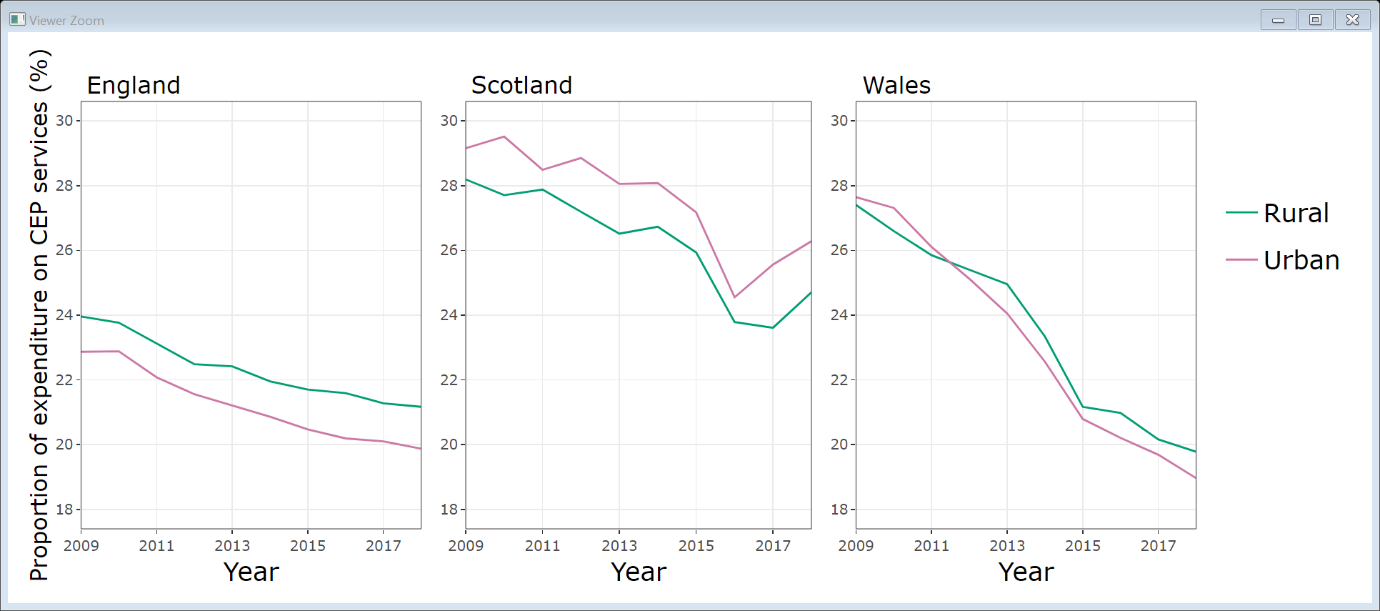


## Appendix 10

Proportion of gross expenditure on CEP services by local government structure in England


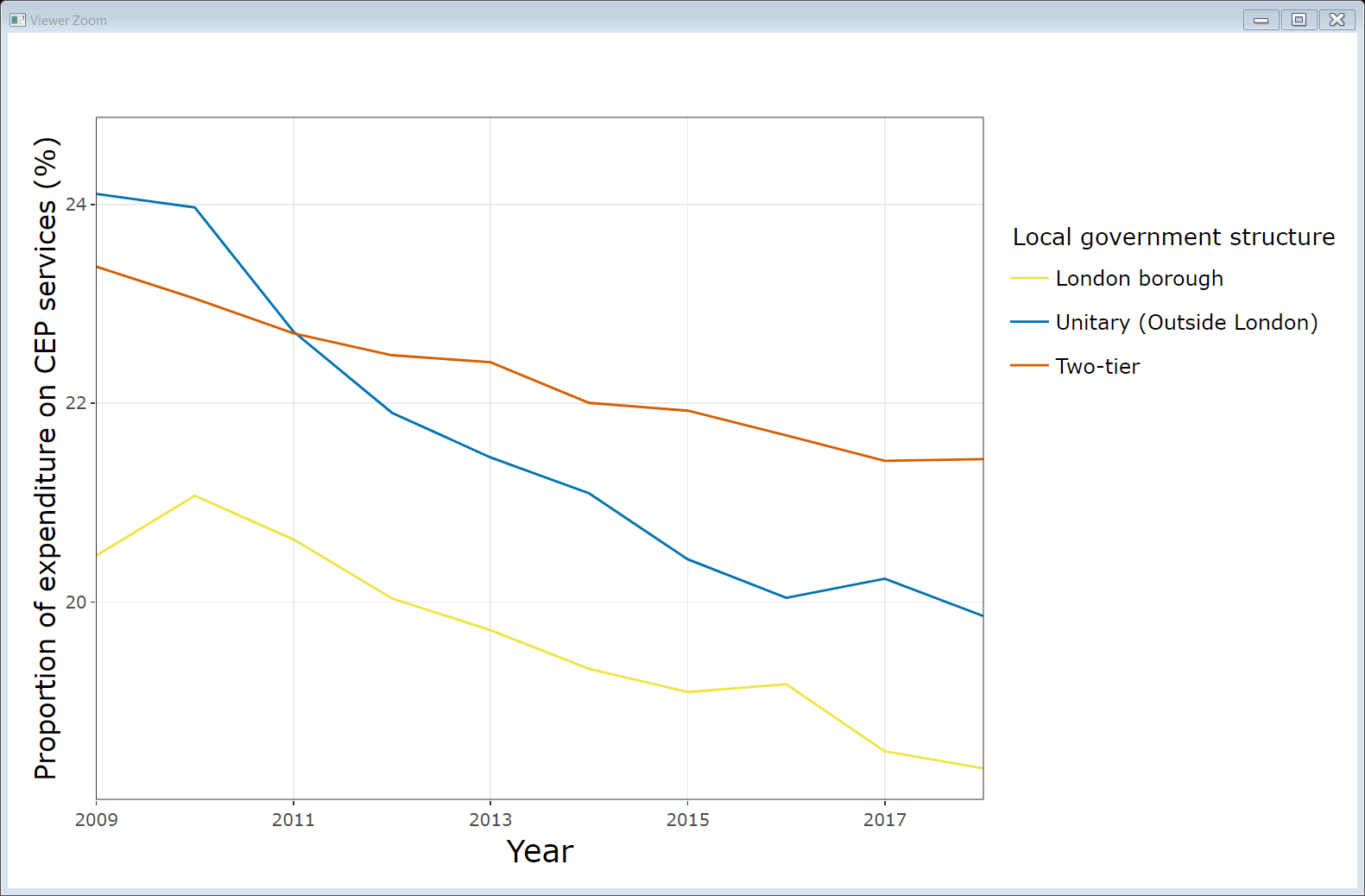


## Appendix 11

Results of Chi-square tests of three-way interactions in generalised estimating equations, comparing nested models.

| Model | P-value |
| --- | --- |
| Per capita |  |
| Including interaction of year, country and deprivation | <.001 |
| Including interaction of year, country and rurality | <.001 |
| Share |  |
| Including interaction of year, country and deprivation | <.001 |
| Including interaction of year, country and rurality | <.001 |

## Appendix 12

| Country | IMD quintile  (1 = least deprived,  5 = most deprived) | Annual percentage change in CEP expenditure per capita (95% CI) | Annual percentage change in CEP share of expenditure (95% CI) |
| --- | --- | --- | --- |
| England | 1 | -4.45 (-5.60, -3.29) | -1.37 (-2.16, -0.57) |
|  | 2 | -5.20 (-6.34, -4.05) | -1.85 (-2.78, -0.92) |
|  | 3 | -5.46 (-6.79, -4.12) | -1.80 (-2.74, -0.85) |
|  | 4 | -5.87 (-6.90, -4.83) | -2.02 (-2.61, -1.42) |
|  | 5 | -7.46 (-8.86, -6.03) | -3.14 (-4.03, -2.24) |
| Scotland | 1 | -5.03 (-5.89, -4.17) | -2.96 (-4.19, -1.71) |
|  | 2 | -3.13 (-4.81, -1.42) | -0.98 (-2.33, 0.38) |
|  | 3 | -4.76 (-5.90, -3.59) | -6.01 (-8.44, -3.51) |
|  | 4 | -5.05 (-5.99, -4.10) | -2.13 (-3.35, -0.89) |
|  | 5 | -3.98 (-5.88, -2.05) | 0.21 (-0.99, 1.42) |
| Wales | 1 | -5.77 (-6.50, -5.04) | -3.95 (-5.01, -2.87) |
|  | 2 | -6.09 (-7.40, -4.77) | -4.04 (-5.43, -2.62) |
|  | 3 | -7.79 (-8.60, -6.98) | -4.83 (-5.44, -4.22) |
|  | 4 | -7.20 (-8.01, -6.38) | -4.52 (-5.29, -3.75) |
|  | 5 | -6.07 (-6.78, -5.36) | -4.86 (-5.70, -4.02) |

## Appendix 13

Population-weighted cartogram of the change in gross CEP expenditure per capita between 2009/10 and 2018/19.


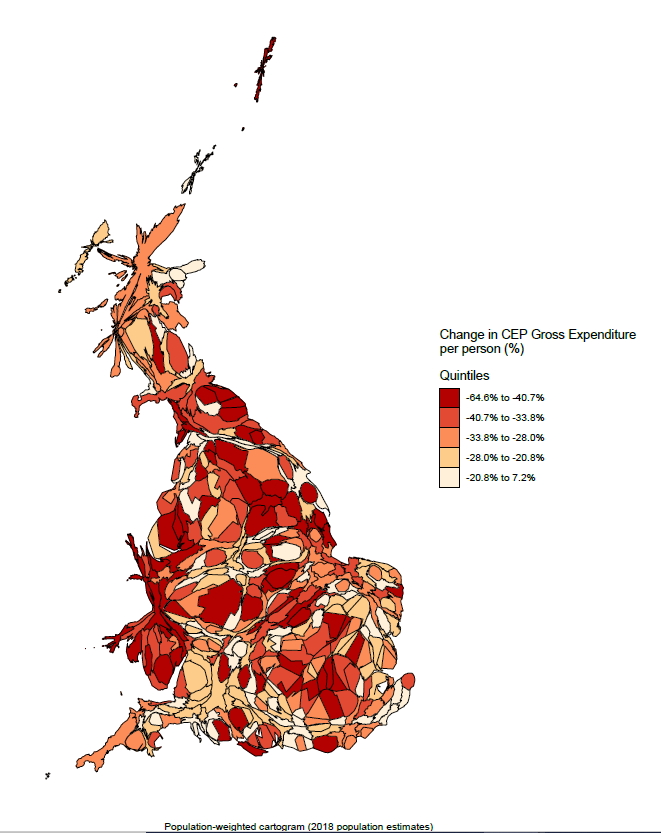


## Appendix 14

Income per capita generated from provision of CEP services between 2009 and 2018, stratified by deprivation quintile in England and Scotland.


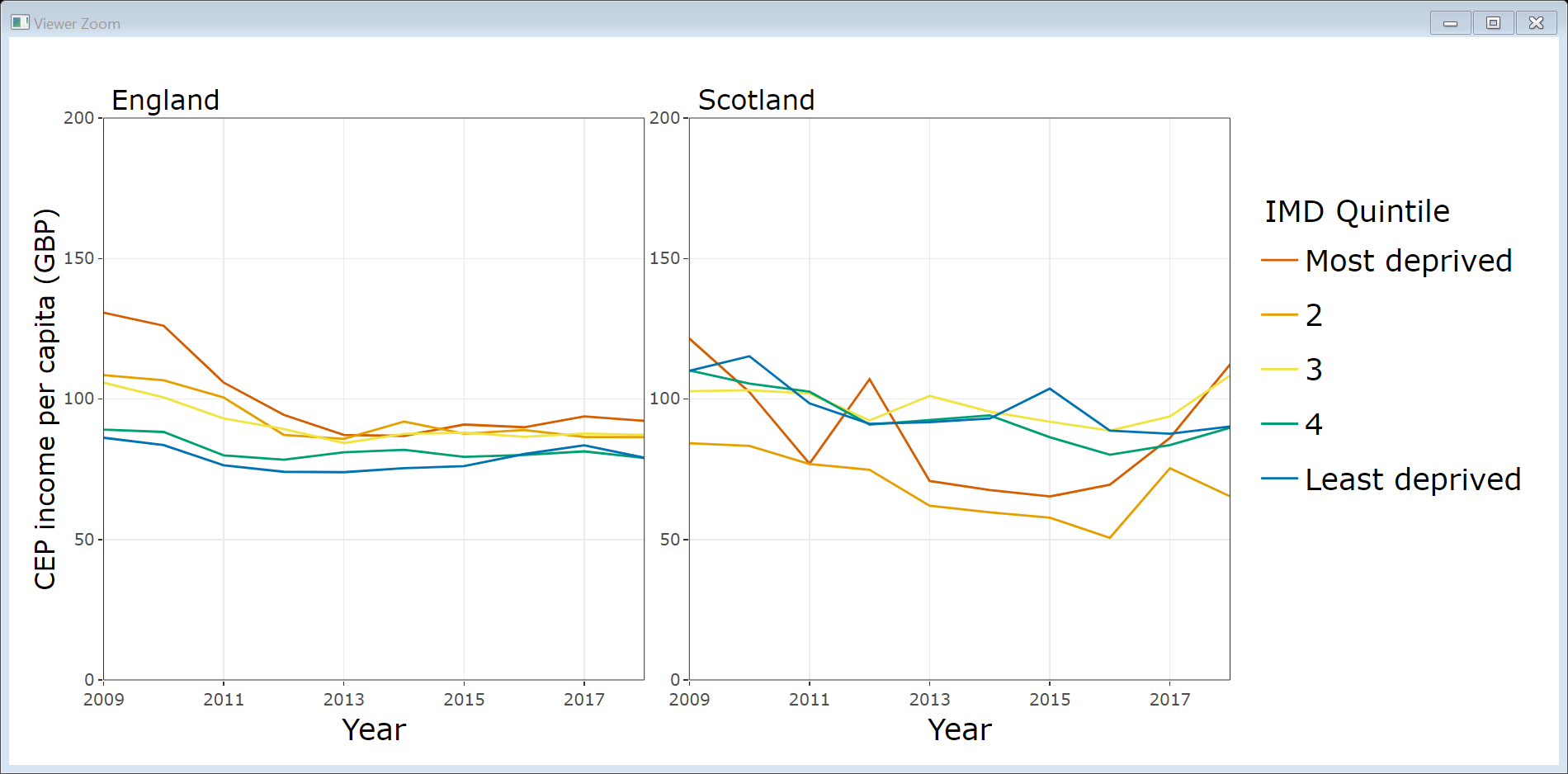

Supplement: Supplementary file 1 — Supplementary Material 1 [file 12889_2023_15179_MOESM1_ESM.docx]
